# Virological Factors Associated with Failure to the Latest Generation of Direct Acting Agents (DAA) and Re-Treatment Strategy: A Narrative Review

**DOI:** 10.3390/v13030432

**Published:** 2021-03-08

**Authors:** Lorenzo Onorato, Mariantonietta Pisaturo, Mario Starace, Carmine Minichini, Alessandra Di Fraia, Roberta Astorri, Nicola Coppola

**Affiliations:** Department of Mental Health and Public Medicine, Section of Infectious Diseases, University of Campania L. Vanvitelli, 80138 Naples, Italy; lorenzoonorato@libero.it (L.O.); mariantonietta.pisaturo@unicampania.it (M.P.); mariostarace1984@libero.it (M.S.); carmine.minichini@alice.it (C.M.); alessandradifraia91@gmail.com (A.D.F.); Roberta.astorri@hotmail.com (R.A.)

**Keywords:** RAS, DAA, virological failure, chronic hepatitis C, retreatment

## Abstract

The availability of all oral direct acting antiviral agents (DAAs) has revolutionized the management of HCV infections in recent years, allowing to achieve a sustained virological response (SVR) in more than 95% of cases, irrespective of hepatitis C Virus (HCV) genotype or staging of liver disease. Although rare, the failure to the latest-generation regimens (grazoprevir/elbasvir, sofosbuvir/velpatasvir, pibrentasvir/glecaprevir) represents a serious clinical problem, since the data available in the literature on the virological characteristics and management of these patients are few. The aim of the present narrative review was to provide an overview of the impact of baseline RASs in patients treated with the latest-generation DAAs and to analyze the efficacy of the available retreatment strategies in those who have failed these regimens.

## 1. Introduction

Hepatitis C Virus (HCV) infection represents one of the most serious threats for health globally. According to the World Health Organization (WHO), 71.1 million people live with HCV infection worldwide, with a total of 1.75 million new cases estimated in 2015 [1]. Approximately 10–20% of chronically infected subjects will develop complications throughout their life, including decompensated cirrhosis and hepatocellular carcinoma [2], which are responsible for about 399,000 deaths every year [1].

Furthermore, unlike what has been observed for other communicable diseases, the mortality rate for viral hepatitis has not decreased over the years, and it is estimated that the number of deaths related to these infections will exceed those due to HIV, tuberculosis and malaria taken together by 2040 [3]. To counteract this worrisome trend, the WHO has proposed an ambitious schedule, based on different strategies, such as the improvement of blood safety, implementation of harm reduction interventions for people who inject drug, and promotion of screening and linkage to care programs among key populations, in order to obtain an 80% decrease in incidence and 65% reduction in mortality of HCV infection by 2030 [4]. However, these goals would never have been achievable without the availability of all oral direct acting antiviral agents (DAAs), which have revolutionized the management of HCV infections in recent years.

The use of highly tolerable and effective combinations currently recommended by international guidelines (third generation DAAs) [5] has allowed the achievement of a sustained virological response (SVR) in more than 95% of cases, irrespective of HCV genotype or staging of liver diseases. However, the presence of a resistance-associated substitution (RAS), particularly in patients who failed a previous DAA-based treatment, still represents an obstacle to achieving a virological cure in a not-negligible number of subjects.

The aim of the present review was to provide an overview of the impact of baseline RAS on the virological response to the latest-generation DAA treatment and to analyze the efficacy of the available re-treatment strategies in patients who have failed the currently recommended regimens.

## 2. HCV Virology

HCV is a positive-sense single-stranded RNA virus [ssRNA (+)], of approximately 65 nm in diameter, roughly spherical and enveloped [6]. It is a member of the Flaviviridae family, sub-grouped in the Hepacivirus genus [7]. The HCV genome consists of a single ssRNA(+) molecule (~9.6 Kb) [8], with conserved untranslated regions (UTRs) at the 5′ and 3′ termini flanking a wide open reading frame (ORF) encoding a large polyprotein, which is cleaved by cellular and viral proteases to produce at least ten proteins [6]: three “structural” proteins: nucleocapsid or core (C) and the two envelope proteins (E1, E2), five “non-structural” proteins (NS3, NS4A, NS4B, NS5A, and NS5B) required for the proper formation of the viral replication complex (RC); two additional proteins, termed p7 and NS2, crucial for virion production and exit, are apparently not involved in viral replication [9,10].

It is worth noticing that genomic RNA lacks both a 5′cap and polyadenylation, and the 5′ and 3′ UTRs are key regulators of viral replication and translation, likely due to peculiar structural organization and rearrangements[11,12]; additional regulatory elements have been identified in the coding regions [13]. Of importance, the NS3 is a multifunctional protein involved in viral replication, immune evasion, polyprotein processing, and using NS4 as a co-factor [10,14]. Differently from other flaviviruses, neutralizing antibodies are elicited by the heavily glycosylated E1 and E2 proteins [15].

Mainly hepatotropic, HCV enters the hepatocyte via receptor-mediated endocytosis, involving partially characterized receptors and co-receptors including CD81, scavenger receptor B1 (SCARB1), tight junction proteins occludin (OLCN) and claudin (CLDN-1), a low-density lipoprotein receptor (LDL-R), epidermal growth factor receptor (EGFR) and more[16]; subsequently, the ssRNA(+) is uncoated, translated and processed in the cell cytoplasm, on the endoplasmic reticulum (ER); guided by signal sequences, the non-structural proteins organize on the ER to form the membrane-bound RC; the NS5B protein represents the RNA-dependent RNA polymerase (RdRp), responsible for the production of a complementary negative-strand intermediate RNA, functioning as a template to synthesize new ssRNA(+) molecules which will be packed within C and E glycoproteins to form mature viral particles, which are included in vesicles and ready to exit the cell via the Golgi-dependent secretory pathway [17]. Thus, a mature HCV particle consists of a nucleocapsid containing the viral RNA, wrapped by an ER-derived lipid bi-layer with E1/E2 heterodimers [6].

Although 7 genotypes, plus a putative eighth [18,19], have been identified, with 90 recognized subtypes up to 2019 [7], the viral RdRp lacks proofreading, in the context of a massive replication within an infected individual, results in genomic instability, with the creation of multiple variants, to be considered a viral “quasispecies”, possibly complicating clinical management [20].

## 3. HCV Treatment

Since 2014, regimens without interferon, which combine several classes of DAAs, well-tolerated and extremely effective have dramatically changed the landscape of HCV therapy, improving the response rate and tolerability.

The DAA regimens allow the eradication of HCV infection even in patients with advanced liver diseases [5,21]. This allows preventing liver complications and HCV-related extrahepatic diseases, improves the quality of life and prevents transmission of HCV [22,23,24].

The surrogate of HCV eradication after therapy is an SVR, defined by undetectable HCV RNA in serum or plasma 12 weeks (SVR12) or 24 weeks (SVR24) after the end of therapy, as assessed by a sensitive molecular method with a lower limit of detection of <15 IU/mL. Both SVR12 and SVR24 have been accepted as endpoints by regulators in Europe and the United States, given that their concordance is >99% [25]. Long-term follow-up studies have shown that an SVR corresponds to a definitive cure of HCV infection in the majority of cases [26,27]. SVR is generally associated with normalization of liver enzymes and improvement or regression of liver necroinflammation and fibrosis, and improvement in liver function [22,23,24].

However, patients with advanced fibrosis (METAVIR score F3) and patients with cirrhosis (F4) who achieve an SVR should remain under surveillance for HCC every 6 months by ultrasound. Long-term post-SVR follow-up studies have shown that the risk of developing HCC remains in patients with cirrhosis who eliminate HCV, although it is significantly reduced compared to untreated patients or patients who did not achieve an SVR [22,28,29,30].

### 3.1. Viral Target and DAAs

Three NS genes, targeted by DAAs in clinical practice, play an essential role for viral replication: NS3/4A, NS5A and NS5B [31]. NS3/4A constitutes a serine protease enabling polyprotein cleavage and maturation [32]. NS5A is a non-enzymatic protein involved in assembly at the cell membrane and replication [33]. Finally, NS5B is an RNA-dependent RNA polymerase and therefore essential for HCV replication [34].

NS3/4A protease inhibitors (PI) were the first DAA to be developed. In general, this DAA class may have a low resistance barrier, multiple drug-drug-interactions due to metabolism via cytochrome P450 and mainly gastro-intestinal side effects. These are the PIs-generations: the first-generation, boceprevir and telaprevir, have now been withdrawn from the market, the second-generation simeprevir (SMV), paritaprevir (PTV), and grazoprevir (GRZ) presented a better efficacy and tolerability profile but active only in genotypes 1 and 4; lastly two pan-genotypic PIs were approved: voxilaprevir (VOX) and glecaprevir (GLE) [35,36,37].

The NS5A inhibitors are characterized by a pan-genotypic activity, by a very low barrier to resistance and show little drug-drug-interactions. There are six approved substances: daclatasvir (DCV), ledipasvir (LDV), ombitasvir (OBV), elbasvir (EBR), velpatasvir (VEL), and pibrentasvir (PIB) [35,36,37]; only the last three substances are currently in use in clinical practice.

NS5B nucleos(t)ide polymerase inhibitors (NS5B-NI) impair the viral replication by providing ”false” substrates for the polymerase, leading to premature chain termination. Sofosbuvir (SOF) is the only pan-genotypic NS5B-NI with high efficacy, resistance barrier, and tolerability. NS5B non-nucleos(t)ide polymerase inhibitors (NS5B-NNI) inhibit NS5B by binding outside the active site, resulting generally in a low barrier to resistance; Dasabuvir (DSV) is the only NS5B-NNI and its use is restricted to genotype 1 [35,36,37].

### 3.2. Treatment Indication and Current Regimens

Table 1 shows the therapeutic options in patients with HCV infection naive to previous DAA treatment according to current guidelines, taking into account genotype, liver disease and previous treatment experience. According to the American Association for the Study of Liver Diseases (AASLD) together with the Infectious Diseases Society of America (IDSA), the European Association for the Study of the Liver (EASL), and the European Aids Clinical Society (EACS), HCV treatment is indicated for all patients with chronic HCV infection, except those with a short life expectancy that cannot be remediated by HCV treatment, liver transplantation, or another directed therapy [5,21,38].

Few are the contraindications to current DAA-based treatments. The use of certain cytochrome P450/P-gp-inducing agents (such as carbamazepine, phenytoin and phenobarbital) contraindicates all DAA regimens, due to the risk of significantly reduced concentrations of HCV DAAs. To date, before starting treatment with a DAA, a complete and detailed drug history should be taken, including all prescribed medications, herbal and vitamin preparations, and any illicit drugs used [5,21,38]. Moreover, it is important to know that treatment regimens comprising an HCV protease inhibitor, such as grazoprevir, glecaprevir or voxilaprevir, are contraindicated in patients with decompensated (Child Pugh B or C) cirrhosis and in patients with previous episodes of decompensation [5,21,38].

## 4. Impact of the Most Frequent RASs on the Virological Response to the latest DAAs

In Table 2, Table 3 and Table 4 we summarized the most frequent RASs, natural or acquired, after a failure to a DAA regimen, in the three target HCV regions according to the lastest-generation DAA and HCV genotype.

The reference amino acid sequence for each HCV genotype was defined as reported by Geno2Pheno. Amino acid substitutions with in-vitro fold-change >2 or found at failure after a specific inhibitor with fold-change unavailable or ≥2 are reported in the Tables.

The natural RASs are frequent in all HCV regions, but especially in the NS5A region. However, the significance of the natural presence of RASs is closely related to the viral genetic background, as they reach a particularly high prevalence in specific subtypes and/or may confer specific levels of resistance.

It is known that a high number (>100) of RASs in NS3, NS5A, and NS5B have been associated in-vivo and/or in-vitro with reduced susceptibility to DAAs; however, not all RASs are equally relevant from a clinical point of view in all HCV-genotypes/subtypes and for all DAAs. We considered RASs relevant if they were described to be associated with treatment failure in vivo and/or if they conferred greater than two-fold changes in drug susceptibility in comparison with a wild-type reference strain in the vitro replicon in assays that were mainly conducted by the industry and published in several studies. [39,40,41,42,43,44,45,46,47,48,49,50,51,52,53,54,55,56,57,58,59,60,61,62,63,64,65,66,67,68,69,70,71,72,73,74].

Several studies have investigated the prevalence of RASs in naïve or treatment-experienced patients and their impact on the virological outcome. In a recent extensive analysis of datasets from various registration trials for the six different NS5A inhibitor-containing regimens, natural ”primary” NS5A RASs (in 4 amino-acid positions 28-30-31-93) have been frequently detected at baseline as natural variants in patients with HCV-genotype (GT) 1a, GT 1b or GT 3, but their impact was often minimized with the use of an intensified treatment regimen, such as a longer treatment duration and/or addition of ribavirin [75]. In 2019, Papaluca et al. [76] assessed the prevalence of RASs in NS5A region among 672 treatment-naïve subjects infected with genotype 1a, 1b or 3 from Australia. The authors demonstrated that 7.6% (25/329), 15.7% (8/51) and 15.1% (44/292) of the patients infected with GT1a, GT1b and GT3, respectively, presented at least 1 substitution at positions 28, 30, 31, 58, 92 or 93, associated to reduced susceptibility to the latest-generation regimens.

As regards the impact of pre-existing RASs on the efficacy of the last DAAs, a recently published multicenter study [77] enrolling 882 German naïve patients infected with genotype 1a, which were included in the European Resistance Database, reported a 6.5% baseline prevalence of RASs conferring high-level resistance to elbasvir; in particular, 3.3% of patients harbored the Q30H/R, 1.8% the L31M and 1.6% the Y93H. Most of them were treated with EBR/GRZ for 16 weeks with ribavirin (6), GLE/PIB (6), LDV/SOF (5), SOF/VEL (2), with an overall SVR rate of 95% (20 out of 21), while 1 of 2 subjects who received EBR/GRZ without RBV presented a virological failure, which underlines the importance of the addition of ribavirin in genotype 1a infected patients with baselines RASs treated with elbasvir/grazoprevir.

These data are consistent with the results of a meta-analysis [78] including eight randomized controlled trials (RCTs) for a total of 1297 genotype 1 infected patients treated with EBR/GRZ; the study demonstrated a suboptimal SVR rate (87.4%, 95% CI 82.3% to 92.5%) among subjects with baseline mutations in NS5A.

Regarding the combination sofosbuvir/velpatasvir, a prospective cohort study [79] conducted in Taiwan on 159 HCV monoinfected and 69 HIV/HCV coinfected patients treated with SOF/VEL reported a 53.5% (122 of 228) baseline prevalence of RASs in the NS5A and 3.1% (7 of 228) in the NS5B region. The authors demonstrated a similar SVR rate among patients with and without substitutions. Only three patients had a virological failure, and two of them harbored no RAS at baseline. However, a more relevant impact of baseline RASs has been demonstrated for genotype 3 patients with advanced liver disease. In 2018, a phase 2 trial [80] randomized 204 cirrhotic subjects infected with genotype 3 to receive either SOF/VEL or SOF/VEL+ribavirin; in the arm without ribavirin a smaller proportion of patients (16 of 19, 84%) with baseline NS5A RASs achieved an SVR12 compared to those without RASs (76 of 79, 96%), while no significant difference in the virological outcome was observed in patients with or without RASs (95% vs. 99%) in the ribavirin arm. In the same year, von Felden et al. [81] demonstrated that the addition of RBV in the case of advanced liver disease minimized the impact of RASs among 293 subjects infected with genotype 3 treated with SOF/VEL, with no virological failure reported among the 22 patients with baseline RASs in the NS5A region.

As regards patients treated with pibrentasvir and glecaprevir, a recently published meta-analysis [82] including 17 RCTs or prospective cohort studies investigated the impact of baseline RASs on the SVR12 rate among 3,302 HCV-infected naïve or experienced patients treated with glecaprevir/pibrentasvir. The study demonstrated that subjects presenting any baseline RAS achieved a lower SVR rate compared to patients without RASs (0.32, 95% CI: 0.15–0.65). The same result was confirmed in the sub-analysis according to the genotypes; the presence of RASs in the NS5a region was associated with treatment failure both among genotype 1 and 3 infected subjects, while a correlation between the presence of any RAS and lower SVR rate was demonstrated in genotype 3, but not in genotype 1. Similar findings were reported in a subsequent meta-analysis including 6501 patients treated with GLE/PIB [83]; again, the study demonstrated that the presence of RASs in any region reduced the SVR rate in genotype 3 patients, while only NS5A substitutions impacted significantly the virological outcomes in genotype 1.

## 5. Retreatment Strategy in Patients Who Failed the Latest DAA Regimens

No randomized controlled study has assessed the best retreatment strategy for patients who failed the latest-generation DAA regimens. The main characteristics of the studies that addressed this issue are shown in Table 5.

### 5.1. Retreatment after Failure to Grazoprevir/Elbasvir

Data on patients’ retreatment following virological failure with GZR/EBR regimens are still very limited, and most of them come from a few observational studies evaluating the effectiveness of the combination sofosbuvir/velpatasvir/voxilaprevir (SOF/VEL/VOX). Hereafter, we report currently available data and observations.

In 2019, Belperio et al. [84] evaluated the efficacy of 12 weeks SOF/VEL/VOX therapy in 573 patients (GTs 1–4) from the Veterans Affairs’ Clinical Case Registry who had previous DAA failure, including 89 GT1 and 1 GT4 infected patients who had experienced EBR/GZR failure and 6 patients with GT1 having failed GZR/EBR + SOF; considering the 87 subjects with available SVR results, among the GZR/EBR experienced patients, 73/80 GT1 and 1/1 GT4 infected patients, along with 4/6 GT1 with prior GZR/EBR + SOF treatment achieved SVR by salvage therapy with only 5 virological failures observed.

Abergel et al. [85] investigated the efficacy of a short-course 8-week GZR/EBR therapy in an international cohort of treatment-naïve subjects with HCV GT1b infection and non-severe liver fibrosis in a phase-3-multicenter study; 112 patients were selected for the analysis: 5 subjects with virological failure were retreated with sofosbuvir/velpatasvir/voxilaprevir for 16 weeks (1), sofosbuvir+glecaprevir/pibrentasvir for 12 weeks (3), or sofosbuvir+glecaprevir/pibrentasvir for 16 weeks(1); all these patients eventually achieved SVR12.

Our group focused on an Italian cohort of HCV patients (GTs 1-4) managed by the CampC network, enrolling 61 subjects with failure to therapy with the latest DAAs [86]. Interestingly, the patients failing elbasvir/grazoprevir showed more frequently the presence of substitutions in at least two regions (95%) compared to those previously treated with sofosbuvir/velpatasvir (43%, *p* < 0.0001) or glecaprevir/pibrentasvir (18%, *p* < 0.0001). Of pertinence, of the 33 patients with GT1b HCV who failed EBR/GZR, 15 patients were retreated with sofosbuvir/velpatasvir/voxilaprevir, all obtaining SVR12, despite the high prevalence of RASs.

Another experience of retreatment with SOF/VEL/VOX in patients who had failed grazoprevir/elbasvir is reported in a prospective multicenter study [87] enrolling 137 Spanish patients previously treated with interferon-free regimens, showing one failure to SOF/VEL/VOX in a cirrhotic genotype 4 subject with a baseline Y93H mutation among the nine patients with previous failure to EBR/GRZ included in the study.

A multicenter study conducted in the US [88] included 19 patients who were retreated with SOF/VEL/VOX after failure to grazoprevir/elbasvir; two patients dropped out before the end of treatment, while the other 17 achieved an SVR12, without no virological failure observed.

Finally, Flamm et al. [89] enrolled 231 DAA-experienced patients who were retreated with GLE/PIB for 8–16 weeks or SOF/VEL/VOX for 12 weeks. A total of 21 patients had a previous failure to grazoprevir/elbasvir and all but one were retreated with SOF/VEL/VOX; only two of them failed to achieve an SVR12, with no virological failure reported.

### 5.2. Retreatment after Failure to Glecaprevir/Pibrentasvir

The main retreatment experiences explored in the literature in patients with a previous failure to velpatasvir/sofosbuvir are reported in Table 5.

One potential retreatment strategy in the patients who failed glecaprevir/pibrentasvir was explored in the MAGELLAN-3 study, a phase IIIb open-label trial assessing the efficacy of glecaprevir/pibrentasvir, associated with sofosbuvir and ribavirin for 12 or 16 weeks in patients previously failing GLE/PIB. The interim analysis on 23 patients [90] reported an overall SVR rate of 96%, with only one relapse at four weeks in a genotype 1 infected cirrhotic patient with two baseline RASs (Y93H and Q30K) in the NS5A region.

Another strategy was evaluated by Pearlman et al. [91], who treated with sofosbuvir/velpatasvir/voxilaprevir for 12 weeks 31 patients previously failing GLE/PIB, obtaining an overall SVR rate of 94%, with two relapses at 4 weeks after treatment. One of them was a non-cirrhotic genotype 3 patient with a baseline A30K mutation, while the other was a cirrhotic subject infected with genotype 1, showing a Y93 variant at baseline.

A multicenter cohort study collected the virological characteristics of 90 patients who failed glecaprevir/pibrentasvir from Germany, Spain and Italy [64]; 59 presented after treatment a RAS in at least one of the three target regions, mostly in NS5A (53, 62.4%). Among the 52 subjects retreated with sofosbuvir/velpatasvir/voxilaprevir, the SVR12 data were available for 46 patients, and only one virological failure (2.2%) was documented in a patient infected with genotype 3, with two baseline RASs in the NS5A region (A30K and Y93H) and two in the NS3 region (Y56H and Q168R). Another four patients were retreated with sofosbuvir/velpatasvir, and three of them (75%) achieved an SVR12.

In 2019 a cohort study [92] including 27 centers in northern Italy evaluated the effectiveness of SOF/VEL/VOX ± RBV in the retreatment of 179 consecutive subjects who had failed DAA-based regimens; all 10 patients previously treated with glecaprevir/pibrentasvir obtained an SVR. The following year, Flamm et al. [89] reported the efficacy of retreatment with SOF/VEL/VOX among four patients with failure to glecaprevir/pibrentasvir; 3 of 4 (75%) achieved an SVR12, with one virological failure in a cirrhotic genotype 1a subject.

### 5.3. Retreatment after Failure to Sofosbuvir/Velpatasvir

The main retreatment experiences explored in the literature in patients with previous failure to velpatasvir/sofosbuvir are reported in Table 5.

In 2019 Belperio et al. [84] reported an SVR rate of 84.4% among 45 patients retreated with SOF/VEL/VOX after failure to sofosbuvir/velpatasvir, with failure rates equally distributed between genotypes.

In a recently published study, Papaluca et al. [93] reported the efficacy of sofosbuvir/velpatasvir/voxilaprevir among 99 NS5A inhibitor-experienced patients with advanced liver disease or severe extra-hepatic manifestations; 19 of these patients presented previous failure to SOF/VEL. Only 1 out of 19 patients (5.3%) with a baseline Y93H mutation in NS5A region did not achieve an SVR12.

In the study conducted by Degasperi et al. in 2019 [92], among the 31 patients who had failed sofosbuvir/velpatasvir, only two (6.5%) presented detectable HCV-RNA at 12 weeks after the end of re-treatment with SOF/VEL/VOX; one of them had a Y93H mutation at baseline, which was not present at failure, while the other patient showed no RAS before or after treatment.

The same year Laneras et al. [87] showed, among the 8 subjects who had failed sofosbuvir/velpatasvir, only one virological failure to SOF/VEL/VOX in a cirrhotic genotype 3 patient with no available resistance profile at baseline. Bacon et al. [88] reported an SVR rate of 95% among 20 patients treated with SOF/VEL/VOX as salvage therapy after failure to SOF/VEL. A similar experience was reported in a German real-world study [94] among 19 patients failing sofosbuvir/velpatasvir who were retreated with SOF/VEL/VOX ± ribavirin for 12 weeks, with an SVR12 rate of 100%. A multicenter cohort study [89] enrolled 28 patients failing velpatasvir/sofosbuvir, who were retreated with SOF/VEL/VOX for 12 weeks (21) or with GLE/PIB (7); 20 (95.2%) and 5 (71.4) achieved an SVR, with three virological failures in patients infected with genotype 1a. Finally, Merli et al. [95] explored retreatment with glecaprevir/pibrentasvir in two cases of HIV/HCV coinfected patients who had experienced a failure to SOF/VEL after an early HCV recurrence with severe hepatitis on a transplanted liver. The first patient was infected with genotype 4d and presented two RASs (Y83H and L28M) in the NS5A region and one (S282T) in the NS5B after failure, while the other was a genotype 1a patient with no detectable RAS at failure. Both were retreated with GLE/PIB for 16 weeks and achieved an SVR 12.

### 5.4. Retreatment after Failure to Sofosbuvir/Velpatasvir/Voxilaprevir

The data available in the literature on retreatment strategies of patients failing sofosbuvir/velpatasvir/voxilaprevir are very limited.

To our knowledge, only one retrospective cohort study has recently addressed this issue [96]; the authors reported an SVR12 rate of 22 patients retreated with different regimens after failure to SOF/VEL/VOX. Fifteen patients received glecaprevir/pibrentasvir plus sofosbuvir and ribavirin, two glecaprevir/pibrentasvir alone, four SOF/VEL/VOX and ribavirin, and one sofosbuvir/velpatasvir and ribavirin. Considering the 19 patients for whom the SVR results were available, 15 achieved an SVR12, two patients reported a virological failure after rescue treatment with SOF/VEL and GLE/PIB+SOF+RBV, respectively (both patients showed after failure a Y93H mutation, which was already present at baseline in one case), while another two died before achieving an SVR. For the remaining three patients, the data on the viral response are still pending.

## 6. Conclusions

A significant correlation has been demonstrated between the presence of defined baseline RASs, particularly in NS5a region, and the occurrence of virological failure in specific settings, such as patients with genotype 1a treated with grazoprevir/elbasvir without ribavirin, genotype 3 subjects with advanced liver disease treated with velpatasvir + sofosbuvir, and genotype 1 and three patients receiving glecaprevir/pibrenstasvir. Unfortunately, limited data, mostly from observational studies with small sample sizes, are available on the retreatment of patients failing last generation DAAs. A thorough evaluation of the RASs emerging from treatment is always recommended to optimize the management of these patients. In the majority of cases, the combination sofosbuvir/velpatasvir/voxilaprevir for 12 weeks has shown to be an effective retreatment strategy. However, considering the absence of new drugs or combinations in the pipeline, in cases with a complex resistance profile the retreatment options are substantially limited to SOF/VEL/VOX for a longer duration with or without the addition of ribavirin, or the combination glecaprevir/pibrentasvir and sofosbuvir ± ribavirin. Further prospective studies are necessary to assess the best strategy in this difficult setting.

## Figures and Tables

**Table 1:** Therapeutic options in patients with HCV infection naive to DAAs regimens.

Genotype	Liver Diseases Stage	Recommended DAA Regimens	Durations in Weeks	References
1a, 1b, 2, 3, 4, 5, 6 1a, 1b, 2, 3, 4, 5, 6 1b 1b	No cirrhosis	Sofosbuvir/velpatasvir Glecaprevir/Pibrentasvir Grazoprevir/elbasvir Ledipasvir/sofosbuvir	12 8 12 12	[5,21] [5,21] [5,21] [21]
1a, 1b, 2, 4, 5, 6 1a, 1b, 2, 4, 5, 6 1b 1b 1a,1b 3	Compensated (Child-Pugh A) cirrhosis	Sofosbuvir/velpatasvir Glecaprevir/Pibrentasvir Grazoprevir/elbasvir for patients without baseline NS5A RASs for elbasvir Ledipasvir/sofosbuvir Sofosbuvir/Velpatasvir Sofosbuvir/velpatasvir/voxilaprevir	12 8 12 12 12 with weight based ribavirin 12	[5,21] [5,21] [5,21] [21] [5,21] [5,21]
any genotype 1, 4, 5, 6 1, 4, 5, 6	Decompensated (Child-Pugh B or C) cirrhosis	Sofosbuvir/Velpatasvir Sofosbuvir/Velpatasvir Ledipasvir/Sofosbuvir Ledipasvir/Sofosbuvir	12 with low initial dose of ribavirin (600 mg, increase as tolerated to weight-based dose) 24 12 with low initial dose of ribavirin (600 mg, increase as tolerated to weight-based dose) 24	[5,21] [5,21] [21] [21]

**Table 2:** RASs in NS3 region with fold-change compared to wild-type replicon according to HCV genotype.

Mutation	Reduced Sensibility to	Genotype	Mean Fold-Change Compared to Wild-Type [Substituted aa, Fold]	References
A156G/T/V	Glecaprevir	1A	T: 1400	[39,40,41]
D/Q168A/V				[39,40]
R155K/I/Q/S/T	Grazoprevir		K: 3–6 Q: 35 T: 10	[39,42,43,44,45,46,47,48,49]
A156L/T/V	Voxilaprevir		L: <2.5 T: 581 V < 2.5	[39,50]
R155G/K/L/T	Grazoprevir	1B	K: 2 T: 10	[39,42,43,44,45,46,47,48,49]
A156T/V			T: 131–280 V: 375	[39,42,43,44,45,49,51,52,53,54]
D168A/E/G/H/K/V/Y			A: 14–30; G: 11; E: 3; H: 52; K: 120; V: 14; Y: 4–8	[43,44,45,46,54,55,56]
Q80K/R	Glecaprevir	3		[39,40]
R155K	Grazoprevir	4	K: 4–6	[39,42,43,44,45,46,47,48,49]
A156S/T			S: 6	[39,40,42,43,44,45,51,52,53,54]
D168A/V				[39,40]

**Table 3:** RASs in NS5B region with fold-change compared to wild-type replicon according to HCV genotype.

Mutation	Reduced Sensibility to	Genotype	Mean Fold-Change Compared to Wild-Type [Substituted aa, Fold (HCV Genotype)]	References
S282R/T	Sofosbuvir	1A	T: 13–9	[39,40,57,58,59,60,61,62,63]
S282G/T		1B	T: 8–10	
S282T		2	T: 3–8 (2A) 16 (2B)	
S282T		3	T: 4	
S282T/C		4	T: 6	
S282T		5	T: 18	
S282T		6	T: 9	

**Table 4:** RASs in NS5A region with fold-change compared to wild-type replicon according to HCV genotype.

Mutation	Reduced Sensibility to	Genotype	Mean Fold-Change Compared to Wild-type [Substituted aa, Fold]	References
M28A/G/S/T	Elbasvir	1A	A: 61–91; G: 71429; T: 15–22	[44,51,52,53,55,56,64,65,66]
Q30D/E/G/H/K/R/Y			D: 1433; E: 56; G: 84; H: 6–8; R: 16–24–125	[44,51,52,53,55,56,64,65,66]
L31F/I/M/V			F: 20–96–131; M: 10–15; V: 1261–125	[51,52,53,55,56,64,65,66,67]
Y93C/H/N/S			C: 11–50; H: 220–351–600; N: 932–1333	[44,51,52,53,56,64,65,66,67,68,69]
H58D	Pibrentasvir			[39,40]
Y93H/N			H: 7; N: 7	[41]
L31F/I/M/V	Velpatasvir		F < 100; I: 4 ; M: 16; V: 68	[70,71,72,73]
Y93C/H/L/N/R/S/T/W			C: 4; H: 609; L: <100; N: 2758; R: 497; S: 64; T: <2,5; W: 999	[70,71,72,73,74]
Y93H	Pibrentasvir	3	H: 2–3	[41]

**Table 5:** Studies evaluating retreatment options after failure to the latest-generation DAAs.

First Name, Year [Ref.]	N. of Patients	Age (Mean, SD)	Males, n. (%)	Genotype Distribution, n. (%)	Cirrhosis, n. (%)	Treatment Failed	Retreatment Regimens	SVR, n (%)
Belperio, 2019 [84]	123	64 (5.6)		1: 94 (76.4) 2: 14 (11.4) 3:13 (10.6) 4: 2 (1.6)		72: GRZ/EBR 6: GRZ/EBR + SOF 45: SOF/VEL	SOF/VEL/VOX 12 weeks	69 (95.8) 4 (66.7) 38 (84.4)
Abergel, 2019 [85]	5			1b: 5 (100)		GRZ/EBR	GLE/PIB + SOF 12 weeks GLE/PIB + SOF 16 weeks SOF/VEL/VOX 16 weeks	3/3 (100) 1/1 (100) 1/1 (100)
Pisaturo, 2020 [86]	21	68 (9.5)	16 (76.2)	1a: 2 (9.5) 1b: 17 (81.0) 3a: 2 (9.5)	6 (28.6)	15: GRZ/EBR 5: SOF/VEL 1: GLE/PIB	SOF/VEL/VOX 12 weeks	15 (100) 5 (100) 1 (100)
LLaneras, 2019 [87]	18			1a: 2 (11.1) 1b: 5 (27.8) 2: 3 (16.7) 3: 4 (22.2) 4: 4 (22.2)	6 (33.3)	9: GRZ/EBR 8: SOF/VEL 1: GLE/PIB	SOF/VEL/VOX 12 weeks	8 (88.9) 7 (87.5) 1 (100)
Bacon, 2019 [88]	40					20 SOF/VEL 19 GRZ/EBR 1 GLE/PIB	SOF/VEL/VOX 12 weeks	19 (95) 17 (89) 1 (100)
Flamm, 2020 [89]	53					21 GRZ/EBR 28 SOF/VEL 4 GLE/PIB	20 SOF/VEL/VOX 12 weeks 1 GLE/PIB 8–16 weeks 21 SOF/VEL/VOX 12 weeks 7 GLE/PIB 8–16 weeks 4 SOF/VEL/VOX 12 weeks	18 (90) 1 (100) 20 (95.2) 5 (71.4) 3 (75)
Wyles, 2020 [90]	23	56 (38–67) *	18 (78)	1: 7 (30.4) 2: 2 (8.7) 3: 14 (60.9)	7 (30)	GLE/PIB	GLE/PIB+SOF+RBV 12–16 weeks	22 (96.0)
Pearlman, 2019 [91]	31		22 (71)	1a: 13 (42) 3a: 18 (58)	18 (58)	GLE/PIB	SOF/VEL/VOX 12 weeks	29 (93.5)
de Salazar, 2020 [64]	50			1a: 13 (26) 1b: 5 (10) 2: 12 (24) 3a: 19 (38) 4d: 1 (2)		GLE/PIB	46 SOF/VEL/VOX ± RBV 12–24 weeks 4 SOF/VEL	42 (91.3) 3 (75)
Degasperi, 2020 [92]	64					31 SOF/VEL 23 GRZ/EBR 10 GLE/PIB	SOF/VEL/VOX ± RBV 12 weeks	29 (93.5) 23 (100) 10 (100)
Papaluca, 2020 [93]	24					19 SOF/VEL ± RBV 3 GRZ/EBR 2 GRZ/EBR + SOF ± RBV	SOF/VEL/VOX 12 weeks	18 (94.7) 3 (100) 2 (100)
Vermehren, 2019 [94]	19					SOF/VEL ± RBV	SOF/VEL/VOX 12 weeks	19 (100)
Merli, 2019 [95]	2	54 (4.2)	2 (100)	1a: 1 (50) 4d: 1 (50)	LT recipient	SOF/VEL	GLE/PIB 16 weeks	2 (100)
Dietz, 2020 [96]	22					SOF/VEL/VOX 12 weeks	13 GLE/PIB + SOF ± RBV 12–24 weeks 2 GLE/PIB 12 weeks 3 SOF/VEL/VOX ± RBV 24 weeks 1 SOF/VEL + RBV 24 weeks	10 (76.9) 2 (100) 3 (100) 0 (0)

* Median, range; LT: Liver Transplantation.

## Data Availability

The datasets used and/or analyzed during the current study are available from the corresponding author on reasonable request.
